# Characterization and Evolution of Volatile Compounds of Cabernet Sauvignon Wines from Two Different Clones during Oak Barrel Aging

**DOI:** 10.3390/foods11010074

**Published:** 2021-12-29

**Authors:** Xu Qian, Fangyuan Jia, Jian Cai, Ying Shi, Changqing Duan, Yibin Lan

**Affiliations:** 1 Center for Viticulture & Enology, College of Food Science and Nutritional Engineering, China Agricultural University, Beijing 100083, China; qianxu@cslg.edu.cn (X.Q.); fangyuansr@163.com (F.J.); shiy@cau.edu.cn (Y.S.); chqduan@cau.edu.cn (C.D.); 2 Key Laboratory of Viticulture and Enology, Ministry of Agriculture and Rural Affairs, Beijing 100083, China; 3 School of Biology and Food Engineering, Changshu Institute of Technology, Changshu 215500, China; 4 Yunnan Engineering Research Center of Fruit Wine, Qujing Normal University, Qujing 655011, China; caijian928@outlook.com

**Keywords:** gas chromatography–mass spectrometry, clone, aroma series, odor activity value, principal component analysis

## Abstract

Xinjiang is a major wine-making region in China, but its hot climate in summer and intense sun exposure negatively affect the aroma quality of Cabernet Sauvignon wine. The aim of this study was to characterize and differentiate the volatile composition of Cabernet Sauvignon wines from two clones (169 and 191) in Xinjiang, and to study their aromatic profile evolution during 12-month oak barrel aging period. Results showed that before aging, clone 169 wine contained higher concentrations of several alcohols and ethyl esters, while acetate esters and furanic compounds were higher in clone 191 wine. After aging, levels of many terpenes, norisoprenoids, volatile phenols and phenolic aldehydes were significantly higher in clone 169 wine than 191 wine. Aroma series analysis revealed that clone 169 wine exhibited higher floral and roasty aromas after aging, while clone 191 wine had stronger chemical aroma. Principal component analysis indicated that aging process played a primary role in the alteration of volatile profile in these wines. Clone played a secondary role and oak barrel had a tertiary contribution to the variation. The present work indicates that clone 169 is a better choice for producing high-quality aged Cabernet Sauvignon wine with intense and elegant aroma in Xinjiang.

## 1. Introduction

Wine has been of particular interest in the field of food sciences due to its unique sensory attributes and health properties [1,2]. Wine aroma is one of the most important aspects that directly affect the overall quality of wine [3,4]. Volatile compounds in wine are mainly derived from three origins: grape berry, fermentation, and aging. Volatile compounds in grapes can be extracted during fermentation process into wine and these volatiles mainly determine the varietal scents of wine [3,5]. Additionally, fermentative aroma of wine, such as higher alcohols and esters, results generally from volatiles generated during the wine fermentation process [6,7]. The wine aging process can further yield volatile compounds that possess aging characteristics, which can further wine complexity [8,9].

There are several volatile compounds derived from grape secondary metabolism, including terpenes, C_13_-norisoprenoids, methoxypyrazines, C_6_ compounds, and volatile phenols [5,10]. These compounds are responsible for the varietal aroma of wines. They are already present in the grapes in free or bound forms, and come into wine through fermentation unaltered or with only minor modifications [5]. Their content in grape is influenced by some genetic factors (variety and clone) and environmental and viticulture conditions (temperature, light exposure, irrigation and nitrogen availability), finally affecting the aroma quality of wine [11]. The typical aromas of certain varietal wines have been associated with some potent volatile compounds. For example, methoxypyrazines such as 3-isobutyl-2-methoxypyrazine (IBMP) and 3-isopropyl-2-methoxypyrazine (IPMP) are responsible for ‘green bell pepper’ aroma in Cabernet Sauvignon, Carmenere, and Sauvignon Blanc wine [12,13], and monoterpenes such as linalool and geraniol greatly contribute to the ‘floral’ character of most Muscat varieties [14]. In addition, several studies have shown that different clones of the same grape variety also exhibit significant differences in their productive characteristics and ability to produce wines with different organoleptic characteristics [15,16,17]. It has been reported that different clones have the ability to produce wines with distinct color [18], phenolic content [19,20,21], aromatic profile [22,23,24,25], and elemental profile [18,26] in several grape varieties. Some research on the differences in volatile composition in grapes and wines from clones of different varieties has been conducted. It has been found that different Sauvignon Blanc clones display varied concentrations of thiol precursors in grape juices and free thiols in wines [27,28]. Belancic et al. (2007) also found that clone was an important factor affecting the methoxypyrazine content in Carmenere wine [12]. Ziegler observed variation in free and bound 1,1,6-trimethyl-1,2-dihydronaphthalene (TDN) and vitispirane concentrations among eight Riesling clones [25]. Therefore, the clonal selection is very important for obtaining the specific characteristics of the grapes and wines.

Oak barrel aging is a common process for red wine maturation since this process can significantly enhance the complexity of wine through incorporating wine with important aging featured volatiles [29,30]. For example, oak barrel odorants, such as lactones and volatile phenols, can be extracted from the oak barrel to the wine during the aging process, which resulting in the aging attributes of red wine [29]. Meanwhile, oxidation, condensation, hydrolysis, and esterification also happen during the wine aging process, potentially resulting in the formation of many new volatile compounds, and these compounds can improve the aromatic complexity of wine [31]. It is known that oak barrel type and toasting level are two important factors that impact wine aging performance [32,33]. It has been reported that a short aging process can release the limited volatiles from the oak barrel to the wine matrix, which does not effectively improve wine aging features. On the other hand, extension of the aging period can break the balance of wine maturation, lowering the wine quality [34].

Several studies have focused on the evolution of different volatile compounds during wine aging in oak barrels [31,35]. However, there are few studies in the literature regarding the behavior of volatile compounds during oak barrel aging of wines from different clones of the same grape variety, such as Cabernet Sauvignon. Xinjiang is a major wine-making region in China, and Cabernet Sauvignon is the primary wine grape variety in this area. However, the high temperature conditions in summer and intense sun exposure have a negative impact on the fruity and floral note, as well as the aroma elegance of Cabernet Sauvignon wine. To improve the overall quality of Cabernet Sauvignon wine produced in this region, it is vital to investigate different grape clones and their impact on wine quality. In the present study, we selected two clones (clone 169 and 191) of Cabernet Sauvignon harvested in 2013 from a commercial vineyard. The same fermentation process took place for both of grape clones and these two wine samples were aged in Wolin and Bordeaux oak barrels for 12 months. This study aimed to characterize and differentiate Cabernet Sauvignon wines from two clones in relation to their volatile composition, and further to study their aromatic profile evolution during the oak barrel aging period. The findings from this study could provide useful information on quality control during the Cabernet Sauvignon wine aging process, as well as important knowledge of the use of clonal selection and evaluation to improve the aroma quality of Cabernet Sauvignon wine produced in the Xinjiang region of China.

## 2. Materials and Methods

### 2.1. Reagents and Chemicals

Volatile standards were purchased from Sigma-Aldrich (St. Louis, MO, USA) and their purity is listed in Table S1. Tartaric acid, dichloromethane (HPLC grade), ethanol (HPLC grade), sodium hydroxide (NaOH), sodium chloride (NaCl), ammonium sulfate ((NH_4_)_2_SO_4_), and sodium sulfate (Na_2_SO_4_) were purchased from Beijing Chemical Works (Beijing, China). Distilled water was purified from a Milli-Q purification system (Millipore, Bedford, MA, USA).

### 2.2. Grape Clones

Cabernet Sauvignon (*Vitis vinifera* L.) clone 169 and 191 vines were grown in a commercial vineyard (Yuanyi Farm) at Manas County (86°12′2″ E and 44°17′55″ N) located in Xinjiang Province of China. This region has semi-arid climate ecological features, with a low annual rainfall, a high average daytime temperature, and a big daytime-to-nighttime temperature difference [36]. These two clone vines were originally planted in 2008. The vines were arranged in north–south rows with a spacing of 3.0 × 1.0 m and were trained to a slope trunk with a vertical shooting positioning trellis system [37]. The horizontal cordon was 0.8 m above ground with a canopy wall 1.5 m high and 0.6 m thick, and each spur pruned cordon had a bud load of 13–15 nodes per linear meter.

Grapes were hand-harvested during 2013 vintage (24 September 2013) in light of their commercial maturity (soluble solids 24–25° Brix, total acidity 5–6 g/L of tartaric acid). The harvested grapes were placed into small baskets and immediately transported to the China International Trust and Investment Corporation (CITIC) Guoan winery in Manas County.

### 2.3. Wine Fermentation

Wine fermentation for each Cabernet Sauvignon clone was performed in duplicate using an industrial-scale stainless steel cylindrical fermenter with a 10,000-L capacity. A cold maceration process took place before the fermentation [6]. In brief, the grape (≈8000 kg) was destemmed, crushed, and then mixed with 60 mg/L sulfur dioxide (SO_2_) in the fermenter. The cold maceration was conducted for 7 days at 8–10 °C. Afterwards, 20 g/hL of yeast L2323 (Lallemand Fermented Beverages, Blagnac, France) was inoculated to initiate alcoholic fermentation. The alcoholic fermentation was carried out at 24–26 °C for 7 days. After the alcoholic fermentation, 6.8 mg/L of lactic acid bacteria LALVIN 31 (Lallemand Fermented Beverages, Blagnac, France) was added for malolactic fermentation for 21 days. After the malolactic fermentation, wine was separated from grape pomace and then 60 mg/L SO_2_ added. Physicochemical parameters of the wine made of the clone 169 and 191 are listed in Table 1.

### 2.4. Oak Barrel Aging

The Wolin (Wolin Company, Yantai, China) and Bordeaux oak barrel (Demptos Company, Yantai, China) with a 225-L capacity were selected for wine aging. The wood of both oak barrels used came from a French tree species (*Quercus petraea*) that had been seasoned in the open air for 2–3 years. These two types of oak barrels were classified as fine grained and at a light toasting level, but with different production standards. For example, the fine grain of the Wolin and Bordeaux oak barrels consisted of growth rings of 1.5–2.0 and 1.0–2.0 mm/year, respectively. The light toasting program of the Wolin oak barrel was 180 °C for 10 min and then 200 °C for 20 min, while for the Bordeaux oak barrel this was 70–80 °C for 25–30 min and then 110–130 °C for 10–15 min. Each clone fermented wine was transferred to both oak barrels in triplicate and kept in a wine cellar with a relative humidity of 70–80% and a temperature of 14–16 °C for 12 months. Wine was sampled in triplicate at the end of malolactic fermentation (0 month), 3, 6, 9, and 12 months of the aging period from each oak barrel. After sampling, the wine was immediately stored at −20 °C before further analysis.

### 2.5. Physicochemical Paremeters

Reducing sugar, ethanol content, pH, volatile acid, and total acidity were measured based on the National Standard of the People’s Republic of China (China, GB/T15038-2006 2006). Reducing sugar of the wine samples was expressed as g/L glucose. Volatile acid and total acidity were expressed as g/L tartaric acid. The physicochemical analysis was performed in triplicate for each sample. Physicochemical parameters of the wine are listed in Table 1.

### 2.6. Headspace Solid-Phase Extraction (HS-SPME)

Headspace solid-phase microextraction for each wine sample was carried out in duplicate according to our previously published methods [38]. The wine sample (5 mL) was mixed with 1 g of NaCl and 10 µL of 1.0018 g/L 4-methyl-2-pentanol (internal standard) in a 20-mL PTFE-silicon septum capped vial. Automatic HS-SPME was performed with a 2 cm DVB/CAR/PDMS 50/30 µm SPME fiber (Supelco, Bellefonte, PA, USA) on a CTC CombiPAL autosampler (CTC Analytics, Zwingen, Switzerland). The SPME fiber was activated at 250 °C prior sample extraction. Afterwards, the vial containing the sample was moved to the heating/stirring equipment and equilibrated at 40 °C for 30 min. The rate of stirring was 500 rpm. Then the SPME fiber was inserted into the headspace of the vial to adsorb volatiles at 40 °C for 30 min under the same stirring rate. Finally, the fiber was removed from the vial headspace and immediately inserted into the GC injection port to fully desorb the volatiles for 8 min.

### 2.7. Liquid–Liquid Extraction

The liquid–liquid extraction for each clone wine was performed in duplicate to extract oak volatile compounds with low volatility and high boiling points. The extraction followed the published methods with minor modifications [22]. The wine sample (20 mL) was mixed with 5 g of (NH_4_)_2_SO_4_ and 40 μL of the internal standards (0.9903 g/L γ-caprolactone, 1.0286 g/L 2-hydroxy-3-methoxybenzaldehyde, and 0.5022 g/L 3,4-dimethylphenol) into a 50-mL centrifuge tube. Afterwards, 5 mL dichloromethane was added to the mixture and then centrifuged at 8000 rpm for 10 min. After centrifugation, the dichloromethane fraction was collected. The aqueous phase was extracted using the same extraction method twice more. The dichloromethane fractions were pooled, dried using Na_2_SO_4_, and concentrated to 1 mL under nitrogen. The final extract was filtered through 0.22 µm organic membrane before gas chromatography–mass spectrometry (GC-MS) analysis.

### 2.8. GC-MS Conditions

An Agilent 6890 GC coupled with an Agilent 5975 Mass Spectrometry (Agilent Technologies, Santa Clara, CA, USA) was used to analyze volatile compounds in these wine samples according to our previously published methods [37,38]. A 60 m × 0.25 mm HP-INNOWAX capillary column with 0.25 μm film thickness (J&W Scientific, Folsom, CA, USA) was used to separate these volatile compounds. Helium was used as the carrier gas and the flow rate was set at 1 mL min^−1^. The injector temperature was kept at 250 °C in the splitless mode (0.75 min). The temperature program of the oven was as follows: 50 °C for 1 min, heated to 220 °C at 3 °C min^−1^ and kept at 220 °C for 5 min. The temperature of the ion source and quadrupole were 250 and 150 °C, respectively. The MSD transfer line heater was set at 250 °C. The electron ionization voltage was set at 70 eV and a full mass scan ranging of *m*/*z* 30–350 was recorded. A C_6_–C_30_ *n*-alkane series (Supelco, Bellefonte, PA, USA) under the same chromatographic conditions was used to calculate retention indices. For the volatiles with their reference standard, volatile compounds were identified by comparing their mass spectrum with the Standard NIST11 library and further confirmed by matching their retention indices with the reference standard. Regarding the volatiles without an available standard, they were tentatively identified by comparing their mass spectrum with the Standard NIST11 library and retention indices with the literature. Quantitation of these volatile compounds in the wine sample followed our published method [6]. Briefly, a synthetic wine matrix (14% ethanol and 5 g/L tartaric acid with pH adjusted to 3.8 using 5 M NaOH solution) was generated based on the average alcohol degree and total acidity of the wine samples in the present study. All the standard solutions were prepared using the synthetic wine matrix, and then diluted to fifteen levels. The extraction and analysis of the standard solution was as the same as the wine sample. For the volatiles with their reference standard, they were quantified using the peak ratio of the reference standard over the internal standard versus the concentration of the reference standard. Regarding the volatiles without an available standard, the quantitation was carried out using the standard that had same carbon atom or similar structure of the volatiles. The concentrations of volatile compounds in both clone wines are listed in Table 2.

### 2.9. Odor Activity Values (OAVs) and Aroma Series

The odor activity value (OAV) is a parameter widely used to evaluate the contribution of individual volatiles to the overall aroma of wine [6,39]. The OAV was calculated as the ratio between the concentration of each volatile compound and its odor threshold [40].

To describe the overall aroma profile of Cabernet Sauvignon wine according to the vast amounts of data obtained from GC-MS analysis, volatile compounds were grouped into 9 aroma series based on similar odor descriptors (Table 3). The 9 aroma series included fruity (1), floral (2), herbaceous (3), sweet (4), spicy (5), fatty (6), chemical (7), roasty (8), and ashy (9). Due to the high complexity of olfactory perception, several volatile compounds were included in two or more aroma series according to some previous studies [6,41]. The intensity of each aroma series was obtained by calculating the sum of the OAVs (∑OAV) of each aroma compound belonging to this series listed in Table 3.

### 2.10. Statistical Analysis

Data were expressed as the mean ± standard deviation. T-test, one-way and two-factor analysis of variance (ANOVA) were used to test the significant difference of the mean at a significant level of 0.05 under Duncan’s test (IBM SPSS Statistics for Windows, Version 20.0, IBM, Chicago, IL, USA). Principal component analysis (PCA) was carried out using MetaboAnalyst (http://www.metaboanalyst.ca/, accessed on 4 August 2021) after normalizing the data via the ‘Autoscaling’ method (mean-centered and divided by the standard deviation of each variable). The heatmap was drafted using ‘pheatmap’ package in R (3.1.0, https://www.r-project.org/, accessed on 4 August 2021) after normalizing the data to clearly show the evolution of various aroma compounds during the aging process.

## 3. Results and Discussion

### 3.1. Volatile Compound Composition

A large dataset of 112 volatile compounds of 120 samples (2 clones × 2 barrels × 5 time points × 6 repetitions) was obtained by GC-MS analysis. A total of 112 volatile compounds were detected in these wine samples (Table 2), which could be classified as: alcohols, esters, fatty acids, terpenes, and norisoprenoids, volatile phenols, furanic compounds, phenolic aldehydes, oak lactones, and other compounds.

#### 3.1.1. Alcohols

**Figure 1:**
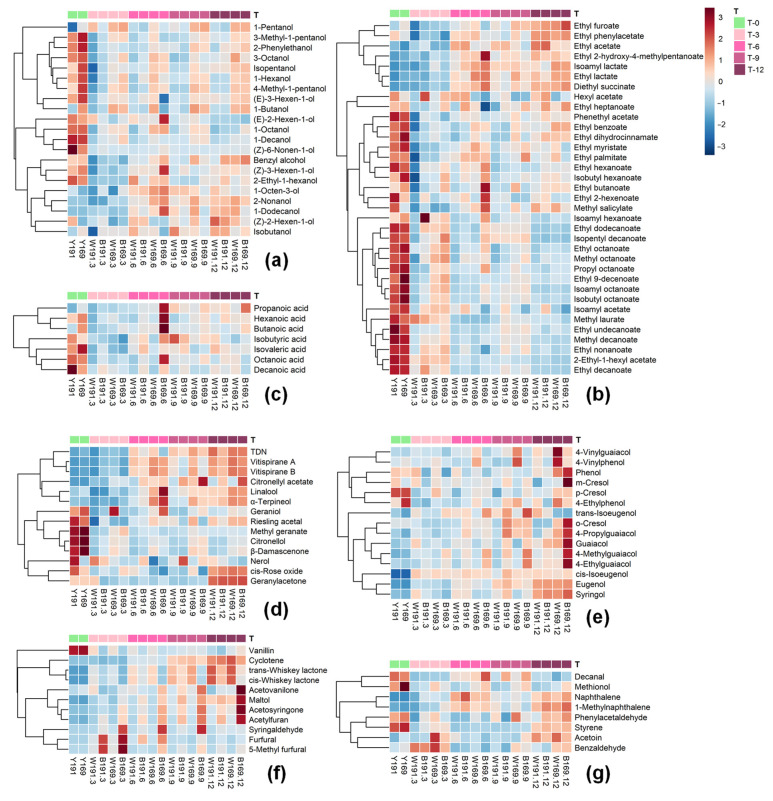
Clustered heatmaps of volatile compounds in wine made of clone 169 and 191 aged in two types of oak barrel during the aging process. T-0, T-3, T-6, T-9, and T-12 represent before aging, 3-month aging, 6-month aging, 9-month aging, and 12-month aging, respectively. Y191 and Y169 represent wine made of clone 191 and 169 before aging, respectively. W191 and W169 represent wine made of clone 191 and 169 aged in Wolin oak barrel for 12 months, respectively. B191 and B169 represent wine made of clone 191 and 169 aged in Bordeaux oak barrel for 12 months, respectively. (**a**) C_6_ alcohols and higher alcohols, (**b**) esters, (**c**) fatty acids, (**d**) terpenes and norisoprenoids, (**e**) volatile phenols, and (**f**) furanic compounds, phenolic aldehydes and oak lactones, (**g**) other compounds.

Alcohols are the major volatile compounds that are yielded during wine fermentation. C_6_ and higher alcohols detected in these wine samples represented about 43% to 73% of the total volatile content, indicating that these volatiles played a significant role in the determination of the overall aroma of these wines. After fermentation, there were five C_6_ alcohols detected in these wine samples, and the clone 169 wine after fermentation exhibited higher total C_6_ alcohols content. Additionally, the individual C_6_ alcohols, including 1-hexanol, (*E*)-3-hexen-1-ol, and (*Z*)-3-hexen-1-ol, were observed to be significantly (*p* ≤ 0.05) higher in the clone 169 wine except for (*Z*)-2-hexen-1-ol (Table 2). During the aging process, a decrease on these C_6_ alcohols happened in both clone wines (Figure 1a). (*E*)-2-Hexen-1-ol, 1-hexanol, (*E*)-3-hexen-1-ol, and (*Z*)-3-hexen-1-ol decreased their content in these wines by 45–52%, 10–12%, 14–22%, and 9–22%, respectively (Table 2). After 12-month aging, the differences in individual C_6_ alcohols between the two clone wines remained unchanged (Table 2).

The clone 169 wine also displayed higher total higher alcohols content compared with the clone 191 wine before the oak barrel aging (Table 2). Most of the individual higher alcohols were present at higher levels in the clone 169 wine than in the clone 191 wine before aging. Clone 169 wine contained significantly (*p* ≤ 0.05) higher concentrations of 1-pentanol, 1-butanol, 3-methyl-1-pentanol, 2-phenylethanol, and isopentanol. 1-Octen-3-ol exhibited significantly (*p* ≤ 0.05) higher levels in the clone 191 wine before the aging process. It has been reported that amino acids in grapes can be metabolized during the wine making process to yield higher alcohols [42]. Therefore, the impact of clonal differences on the higher alcohols levels in these wines before aging might be mainly due to the difference in amino acid composition between these grape clones. Most of the higher alcohols, such as isopentanol, 4-methyl-1-pentanol, 3-methyl-1-pentanol, 1-octanol, and 1-decanol, exhibited a dramatic decrease between 0 and 3 months of the aging process (Figure 1a). Afterwards, the content of these higher alcohols in the wines remained constant or slightly increased until the end of the aging. During the aging period, the concentrations of 1-octanol, 1-decanol, 4-methyl-1-pentanol, and 3-methyl-1-pentanol decreased by 67–70%, 30–40%, 17–21%, and 15–19%, respectively (Table 2). On the contrary, the concentrations of 1-pentanol, 1-octen-3-ol, 2-nonanol, and 1-dodecanol increased by 14–38%, 10–29%, 37–50%, and 48–108%, respectively (Table 2). It has been reported that esters in wine can be hydrolyzed into higher alcohols during the aging process [9]. We speculated that the accumulation of these higher alcohols in the wines during the aging process might result from the hydrolysis of their corresponding esters. At the end of the aging period, the wine made from clone 169 showed significantly (*p* ≤ 0.05) higher content of nine higher alcohols. Regarding these higher alcohols, 1-butanol, 1-pentanol, isopentanol, and 3-methyl-1-pentanol showed higher content in the clone 169 wine before and after the aging process, whereas 3-octanol, 1-decanol, 4-methyl-1-pentanol, and benzyl alcohol were present in a higher content in the clone 169 wine after 12 months of aging. It should be noted that the aging process reduced the content of isobutanol, 2-phenylethanol, and 1-octen-3-ol in the clone 169 wine, making the contents of these higher alcohols similar in both clone wines. The benzyl alcohol content was significantly (*p* ≤ 0.05) higher in the wine aged in the Bordeaux oak barrel than that aged in the Wolin oak barrel after the aging process (Table 2).

#### 3.1.2. Esters

Esters have normally been considered the primary source of the fruity scents and these volatiles could contribute wine with the fruity aroma [7,43]. A total of 35 esters were found in both grape clone wines before aging (Table 2). However, content differences were observed for 10 individual esters. The wine made of clone 169 showed significantly (*p* ≤ 0.05) higher concentrations of ethyl lactate, isoamyl lactate, and ethyl 9-decenoate, whereas ethyl 2-hexenoate, ethyl acetate, hexyl acetate and methyl salicylate were present at significantly (*p* ≤ 0.05) higher levels in the clone 191 wine before the aging process. The aging process significantly reduced the concentration of most of the esters in all wines (Figure 1b). The contents of ethyl 9-decenoate, ethyl decanoate, ethyl benzoate, 2-ethyl-1-hexyl acetate, and isoamyl acetate decreased in all wines by 72–81%, 50–51%, 18–32%, 85–90%, and 10–29%, respectively (Table 2). The decreased concentrations of these esters might mainly result from their hydrolytic reaction during the aging period [44]. Additionally, the oak barrel could absorb esters, resulting in a decrease in the content of these esters [45,46]. It is worth noting that esters are the major volatiles that incorporate fruity and floral notes to the wine aroma, and their concentration decrease during aging might lower the fruitiness of aged wine [9]. In addition, during the aging period, esterification and transesterification also take place, which might lead to increased ester concentration in wine [29,44,47,48]. In the present study, it was observed that seven esters showed an increase on their concentration in both clone wines during the aging process. Ethyl lactate, diethyl succinate, isoamyl lactate, and ethyl acetate increased their concentrations by 311–797%, 357–519%, 388–506%, and 87–100%, respectively. As shown in Table 2, there were significant differences (*p* ≤ 0.05) in 18 esters between the clone 169 and 191 wines after an aging period of 12 months. Among these esters, the differences in ethyl acetate, ethyl butanoate, ethyl lactate, ethyl 9-decenoate, and ethyl 2-hexenoate between the two clone wines were consistent both before and after the aging process. Additionally, the aging process resulted in differences in 2-ethyl-1-hexyl acetate, isoamyl acetate, ethyl benzoate, and isoamyl octanoate between these two clone wines. Furthermore, the wine aged in the Wolin oak barrel had significantly (*p* ≤ 0.05) higher concentrations of ethyl undecanoate and ethyl myristate than the wine aged in the Bordeaux oak barrel after aging.

#### 3.1.3. Fatty Acids

Fatty acids can be yielded during the fermentation process by the activity of yeasts, and these volatile compounds can contribute fatty, pungent, rancid, fruity, and cheesy notes to the overall aroma of the wine [9,39,49]. Before the aging process, seven volatile fatty acids were detected in all wines and their concentrations were similar between the two clone wines except for hexanoic acid and butanoic acid (Table 2). During the aging process, obvious decreases in decanoic acid, octanoic acid, isovaleric acid, isobutyric acid, and hexanoic acid were observed in both clone wines (Figure 1c), which is consistent with previous studies [29,31]. We speculated that their decreases might result from the adsorption of the oak wood [31]. In contrast, a short-chain fatty acid, propanoic acid, displayed an increasing trend in all wines during the oak barrel aging period, which might mainly be attributed to the hydrolysis of their corresponding esters [48]. After 12-month aging, only isovaleric acid showed a significantly (*p* ≤ 0.05) higher level in the clone 191 wine than the clone 169 wine, while the concentrations of other volatile fatty acids were similar between the two clone wines (Table 2). In addition, the wine aged in the Bordeaux oak barrel had a significantly (*p* ≤ 0.05) higher concentration of propanoic acid, whereas the Wolin barrel aged wine had a significantly (*p* ≤ 0.05) higher content of decanoic acid after aging.

#### 3.1.4. Terpenes and Norisoprenoids

Terpenes and norisoprenoids in wine are mainly derived from grape, and these volatile compounds are extracted into wine during the fermentation process [14]. Although terpenes and norisoprenoids are present in wine at relatively low levels, their low odor thresholds mean that these volatiles significantly contribute their flavor notes to the overall aroma of wine [50]. It has been confirmed that terpenes and norisoprenoids possess floral and fruity scents, and the composition of terpenes and norisoprenoids in wine can indicate the varietal features of wines made of different grape varieties [51]. In the present study, a same terpene and norisoprenoid compositions (nine terpenes and five norisoprenoids) were found in both clone wine samples before the aging process (Table 2). Meanwhile, these volatiles, except for *cis*-rose oxide, also exhibited the similar concentrations in these wines. During the aging period, the concentrations of citronellol, *β*-damascenone, and riesling acetal were significantly decreased in these wines, by 62–70%, 46–55%, and 11–31%, respectively (Figure 1d). The decreased concentrations of these volatiles might be the result of acid-catalyzed rearrangements [8,29]. However, increased concentration of vitispirane A (241–280%), 1,1,6-trimethyl-1,2-dihydronaphthalene (TDN, 161–224%), citronellyl acetate (109–267%), vitispirane B (156–188%), and linalool (16–18%) were observed in these wines during the aging process. It should be noted that the concentration of *cis*-rose oxide increased in the clone 169 wine but decreased in the clone 191 wine during the oak barrel aging period. After an aging process of 12 months, the clone 169 wines contained significantly (*p* ≤ 0.05) higher concentrations of three terpenes (linalool, citronellyl acetate, and citronellol) and four norisoprenoids (*β*-damascenone, riesling acetal, vitispirane A, and vitispirane B) (Table 2). In addition, the wines aged in the Bordeaux oak barrel showed significantly higher levels of citronellyl acetate after the aging process than the wines in the Wolin oak barrel.

#### 3.1.5. Volatile Phenols

Volatile phenols are known to be one of the most important volatiles for incorporating aging flavor into the overall aroma of wine during the aging process [10]. Both clone wines before the aging process exhibited the same volatile phenol composition (15 volatile phenols), and no significant differences were observed with respect to their concentrations between the two clone wines (Table 2). As illustrated in Figure 1e, the aging process resulted in a significant accumulation of most volatile phenols in these wine samples, and after 12 months of aging, 12 volatile phenols displayed significant concentration differences in these two clone wines (Table 2). Clone 169 wines after the aging process contained significantly (*p* ≤ 0.05) higher concentrations of guaiacol, phenol, 4-propylguaiacol, 4-vinylphenol, 4-vinylguaiacol, 4-ethylphenol, and 4-ethylguaiacol compared to the clone 191 wines. The variation of volatile phenols between these wine samples after the aging process may be attributed to the different levels of hydroxycinnamic acids precursors present in the grapes [52]. 4-Vinylguaiacol and 4-vinylphenol have been reported to be metabolized from ferulic acid and *p*-coumaric acid, respectively, under the activity of microorganisms, and then reduced to ethyl derivatives (4-ethylguaiacol and 4-ethylphenol) by enzyme vinylphenol reductase [31,52]. On the other hand, it has been reported that the use of oak barrels could enhance the levels of volatile phenols in wine, since they can be extracted from the oak barrel into the wine during aging [34,53]. Oak barrel type, oak toasting level, and wine oak barrel aging period essentially determine the concentrations of volatile phenols in wine [32,53,54,55]. In the present study, it was observed that the wine samples aged in the Bordeaux oak barrel contained significantly (*p* ≤ 0.05) higher levels of guaiacol, 4-methylguaiacol, 4-ethylguaiacol, and *m*-cresol, whereas the Wolin oak barrel resulted in wines with significantly higher levels of 4-ethylphenol, 4-vinylguaiacol, *trans*-isoeugenol, and 4-vinylphenol (Table 2). These results indicated that the Bordeaux oak barrel might be abundant in guaiacol, 4-methylguaiacol, 4-ethylguaiacol, and *m*-cresol, whereas 4-ethylphenol, 4-vinylguaiacol, *trans*-isoeugenol, and 4-vinylphenol might be the major volatile phenols in the Wolin oak barrel.

#### 3.1.6. Furanic Compounds, Phenolic Aldehydes, and Oak Lactones

Like volatile phenols, furanic compounds, phenolic aldehydes, and oak lactones are also important volatile compounds that can be extracted from the oak barrel into the wine during the aging process, and these volatiles can help maturate wine by enhancing the aging aroma of the wine [34]. In the present study, five furanic compounds, four phenolic aldehydes, and two oak lactones were found in these wines. Before the aging process, only furfural and acetylfuran exhibited significantly (*p* ≤ 0.05) higher concentrations in the clone 191 wine than in the clone 169 wine, whereas the other volatiles showed similar levels in the two clone wines. With the aging process, the concentrations of these volatiles increased in all wines, which was a result of the extraction from the oak barrel to the wines (Figure 1f). After an aging process of 12 months, the clone 169 wines showed significantly (*p* ≤ 0.05) higher concentrations of acetylfuran, vanillin, acetosyringone, and acetovanilone than the clone 191 wines (Table 2). Additionally, the wines aged in the Bordeaux oak barrel contained significantly higher levels of furfural, 5-methyl furfural, acetylfuran, acetovanilone, syringaldehyde, and acetosyringone, whereas higher levels of the two oak lactones were found in the wines aged in the Wolin oak barrel after the aging period.

#### 3.1.7. Other Compounds

Both clone wines were found to contain four carbonyl compounds (acetoin, benzaldehyde, benzeneacetaldehyde and decanal), three benzene derivatives (styrene, naphthalene, and 1-methylnaphthalene), one pyrazine (3-isobutyl-2-methoxypyrazine), and one sulfur compound (methionol). Before the aging process, only acetoin and methionol exhibited significantly (*p* ≤ 0.05) higher concentrations in the clone 169 wine, whereas similar concentrations of the other volatiles were found between the two clone wines (Table 2). After 12-month aging, only benzaldehyde was present at a significantly (*p* ≤ 0.05) higher level in the clone 169 wine than the clone 191 wine. Additionally, the wines aged in the Wolin oak barrel had a significantly (*p* ≤ 0.05) higher level of acetoin than the wines aged in the Bordeaux oak barrel.

### 3.2. Principal Component Analysis (PCA)

**Figure 2:**
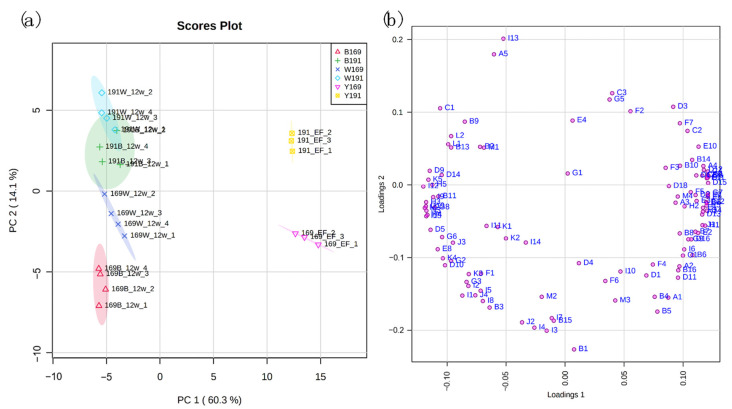
(**a**) Score plot and (**b**) loading plot of principal component analysis using the detected volatiles as the variables. Y191 and Y169 represent wine made of clone 191 and 169 before aging, respectively. W191 and W169 represent wine made of clone 191 and 169 aged in Wolin oak barrel for 12 months, respectively. B191 and B169 represent wine made of clone 191 and 169 aged in Bordeaux oak barrel for 12 months, respectively. The codes of volatile compounds in the loading plot refer to Table S1.

To elucidate the effect of clone and oak barrel type on the evolution of volatile compounds in these wine samples during the aging process, principal component analysis (PCA) was carried out using all the detected volatiles in young and 12-month aged wines as the variables (Figure 2). Principal components 1 and 2 (PC1 and PC2) accounted for 60.3% and 14.1% of total variability, respectively. An obvious segregation was observed between the young and aged wines, positioned at the right and left side of the plot, respectively (Figure 2a). This indicates that the variation of volatile compounds in these wines was mainly due to aging. As seen from the loading plot in Figure 2b, the young wines were mainly characterized by higher levels of numerous esters, including ethyl decanoate, methyl decanoate, ethyl dodecanoate, isobutyl octanoate, isoamyl octanoate, 2-ethyl-1-hexyl acetate, ethyl nonanoate, isopentyl decanoate, ethyl octanoate, etc., while the aged wines were associated with higher levels of volatile phenols (*cis*-isoeugenol, syringol, eugenol, and guaiacol), furanic compounds (furfural, 5-methyl furfural, acetylfuran, and maltol), esters (diethyl succinate, ethyl lactate, isoamyl lactate), *cis-* and *trans*-whiskey lactone, and norisoprenoids (vitispirane A, vitispirane B, and TDN). The second major cause of the variation, along PC2 was the clone (Figure 2a). It was also observed that whether before or after aging, the clone 191 and 169 wines tended to cluster into separate groups on the positive and negative y-axis, respectively (Figure 2a). The corresponding loading plot (Figure 2b) showed that clone 191 wines were characterized by higher levels of some alcohols (1-octen-3-ol and (*Z*)-2-hexen-1-ol), esters (hexyl acetate, ethyl acetate, isoamyl acetate, ethyl 2-hexenoate and methyl salicylate), acids (decanoic acid and isobutyric acid), *trans*-isoeugenol, and nerol. The clone 169 wines were associated with higher levels of several alcohols (1-hexanol, 1-butanol, 1-pentanol, benzyl alcohol and 3-methyl-1-pentanol), volatile phenols (*o*-cresol, *m*-cresol, phenol, 4-propylguaiacol, guaiacol, 4-ethylguaiacol, and 4-methylguaiacol), acetovanilone, acetosyringone, benzeneacetaldehyde, and benzaldehyde. Compared to the variation on the volatile profile caused by the aging and clone, the use of different barrels led to a more subtle difference (Figure 2a).

### 3.3. Aroma Profile Analysis

Table 3 lists the volatile compounds in these wines with OAVs above 0.1, indicating that these volatiles played important roles in affecting the overall aroma of these wines [6]. Furthermore, these major volatiles were grouped into seven aroma series according totheir aroma descriptors, including fruity, floral, sweet, spicy, fatty, chemical, and roasty aroma (Table 3).

**Figure 3:**
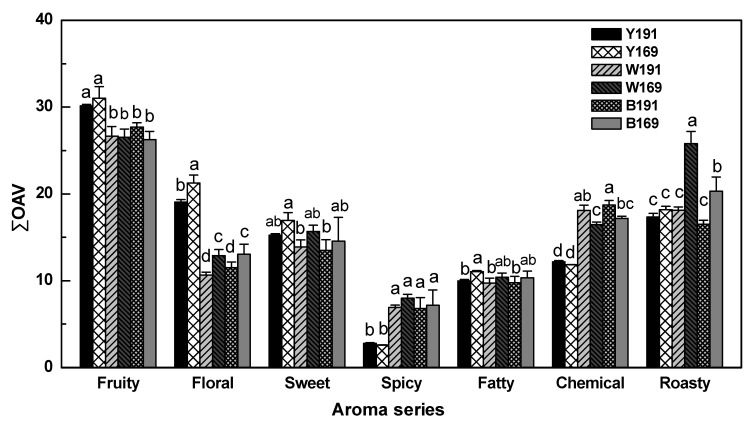
Total odor activity values of aroma series in wine made of Cabernet Sauvignon clone 169 and 191 before and after aging process. Y191 and Y169 represent wine made of clone 191 and 169 before aging, respectively. W191 and W169 represent wine made of clone 191 and 169 aged in Wolin oak barrel for 12 months, respectively. B191 and B169 represent wine made of clone 191 and 169 aged in Bordeaux oak barrel for 12 months, respectively. Different letters indicate significant differences between samples for each aroma attribute at a significant level of 0.05.

It can be observed that fruity and floral aroma series were the main aromatic features of both clone wines before aging (Figure 3). The major volatiles that contributed the fruity note to these wines included *β*-damascenone, ethyl acetate, ethyl hexanoate, and isoamyl acetate, whereas *β*-damascenone, 2-phenylethanol, and geraniol mainly provided the wines with a floral note (Table 3). No fruity aroma variation was observed between the clone 169 and 191 wine before and after 12-month aging (Figure 3). Meanwhile, the clone 169 wines always exhibited higher floral aroma than the clone 191 wines, both before and after aging. Nevertheless, the aging process consistently led to a significant decrease in both fruity and floral aromas. The chemical aroma was mainly contributed by ethyl acetate, isopentanol, and guaiacol in the wines, while volatile phenols including 4-vinylphenol, *p*-cresol, eugenol, guaiacol, and 4-vinylguaiacol played important roles in providing the roasty and spicy notes to the wine (Table 3). Before aging, no significant differences in chemical, roasty and spicy aromas were observed between the clone 169 and 191 wines (Figure 3). Through the aging process, these aged aromas were significantly accumulated in these wines. The clone 191 wines after 12 months of aging exhibited higher chemical aroma than the clone 169 wines, while the clone 169 wines showed much higher roasty aroma after aging (Figure 3). In addition, the sweet aroma in these wines mainly comprised *β*-damascenone, eugenol, and *trans*-whiskey lactone, while fatty acids, isopentanol, ethyl lactate, and ethyl decanoate determined the fatty aroma to these wines (Table 3). The clone 169 wines before aging showed a slightly higher value of fatty aroma compared to the clone 191 wines. However, the aging process resulted in these wines having similar sweet and fatty aromas.

**Table 3:** Odor activity value (OAV), aroma descriptor, and aroma series of main volatile compounds in wine made of Cabernet Sauvignon clone 169 and 191 before and after 12 months of aging.

Volatile	Aroma Threshold	Aroma Descriptor	Aroma Series ^a^	OAV (Before Aging)		OAV (12-Month Aging)			
	(μg/L)			Clone 191	Clone 169	Clone 191 (Wolin)	Clone 169 (Wolin)	Clone 191 (Bordeaux)	Clone 169 (Bordeaux)
***C_6_ alcohols***									
1-Hexanol	1100	Herbaceous, grass, woody	3 [6]	2.12 ± 0.01 ^b^	2.31 ± 0.04 ^a^	1.87 ± 0.13 ^c^	2.05 ± 0.08 ^b^	1.88 ± 0.04 ^c^	2.08 ± 0.06 ^b^
***Higher alcohols***									
Isobutanol	75,000	Alcohol, solvent, green, bitter	3,7 [6]	0.53 ± 0.01 ^c^	0.56 ± 0.00 ^b^	0.59 ± 0.01 ^a^	0.55 ± 0.01 ^b^	0.59 ± 0.01 ^a^	0.57 ± 0.02 ^b^
Isopentanol	60,000	Solvent, alcohol, nail polish	6,7 [6]	3.44 ± 0.02 ^bc^	3.89 ± 0.01 ^a^	3.35 ± 0.11 ^c^	3.49 ± 0.04 ^b^	3.36 ± 0.02 ^c^	3.51 ± 0.05 ^b^
3-Methyl-1-pentanol	500	Pungent, solvent, green	3,7 [6]	0.25 ± 0.00 ^b^	0.32 ± 0.01 ^a^	0.21 ± 0.01 ^c^	0.26 ± 0.01 ^b^	0.21 ± 0.01 ^c^	0.26 ± 0.01 ^b^
1-Octen-3-ol	20	Mushroom	9 [6]	0.37 ± 0.01 ^b^	0.31 ± 0.01 ^c^	0.42 ± 0.04 ^a^	0.38 ± 0.01 ^ab^	0.40 ± 0.03 ^ab^	0.39 ± 0.01 ^ab^
2-Phenylethanol	14,000	Roses, honey	2 [6]	2.31 ± 0.09 ^b^	2.75 ± 0.03 ^a^	1.35 ± 0.11 ^d^	1.78 ± 0.28 ^c^	1.72 ± 0.34 ^cd^	1.96 ± 0.33 ^bc^
***Acetate esters***									
Ethyl acetate	12,270	Pineapple, varnish, balsamic	1,7 [56]	8.61 ± 0.09 ^d^	6.82 ± 0.13^e^	16.61 ± 0.55 ^b^	13.05 ± 0.18 ^c^	17.25 ± 0.54 ^a^	12.74 ± 0.15 ^c^
Isoamyl acetate	160	Banana	1 [6]	2.80 ± 0.03 ^a^	2.89 ± 0.15 ^a^	2.29 ± 0.10 ^c^	2.20 ± 0.08 ^cd^	2.53 ± 0.11 ^b^	2.04 ± 0.14 ^d^
***Ethyl esters***									
Ethyl butanoate	400	Banana, pineapple, strawberry	1 [6]	0.54 ± 0.01 ^bc^	0.59 ± 0.02 ^a^	0.52 ± 0.02 ^c^	0.55 ± 0.01 ^b^	0.54 ± 0.03 ^bc^	0.54 ± 0.01 ^bc^
Ethyl hexanoate	80	Banana, green apple	1 [6]	5.14 ± 0.06 ^a^	4.91 ± 0.31 ^a^	3.99 ± 0.25 ^b^	4.15 ± 0.18 ^b^	4.19 ± 0.22 ^b^	4.20 ± 0.08 ^b^
Ethyl lactate	100,000	Strawberry, raspberry, buttery	1,6 [41]	0.22 ± 0.00^e^	0.53 ± 0.02 ^d^	1.97 ± 0.15 ^bc^	2.20 ± 0.10 ^ab^	1.72 ± 0.26 ^c^	2.27 ± 0.25 ^a^
Ethyl octanoate	580	Sweet, floral, fruity, banana, pear	1,2 [6]	0.96 ± 0.00 ^b^	1.04 ± 0.07 ^a^	0.62 ± 0.05 ^c^	0.67 ± 0.03 ^c^	0.67 ± 0.02 ^c^	0.65 ± 0.01 ^c^
Ethyl decanoate	200	Fruity, fatty	1,6 [6]	1.68 ± 0.00 ^a^	1.64 ± 0.13 ^a^	0.84 ± 0.03 ^b^	0.82 ± 0.02 ^b^	0.83 ± 0.01 ^b^	0.80 ± 0.01 ^b^
Ethyl dihydrocinnamate	2	Sweet, caramel	4 [57]	0.36 ± 0.02 ^b^	0.51 ± 0.08 ^a^	0.16 ± 0.03 ^d^	0.24 ± 0.04 ^c^	0.20 ± 0.07 ^cd^	0.23 ± 0.02 ^cd^
Diethyl succinate	100,000	Over-ripe, lavender	1,2 [41]	0.02 ± 0.00 ^b^	0.03 ± 0.00 ^b^	0.13 ± 0.01 ^a^	0.14 ± 0.01 ^a^	0.14 ± 0.02 ^a^	0.15 ± 0.02 ^a^
***Other esters***									
Isoamyl octanoate	125	Sweet, fruity, cheese, cream	1,4,6 [6]	0.29 ± 0.01 ^b^	0.34 ± 0.04 ^a^	0.13 ± 0.01 ^c^	0.14 ± 0.01 ^c^	0.13 ± 0.00 ^c^	0.13 ± 0.00 ^c^
***Fatty acids***									
Propanoic acid	8100	Pungent, rancid, soy	6 [6]	0.09 ± 0.01 ^c^	0.12 ± 0.00 ^b^	0.12 ± 0.01 ^b^	0.12 ± 0.01 ^b^	0.14 ± 0.02 ^ab^	0.16 ± 0.02 ^a^
Isobutyric acid	2300	Rancid, butter, cheese	6 [6]	0.35 ± 0.01 ^a^	0.30 ± 0.05 ^abc^	0.31 ± 0.03 ^ab^	0.24 ± 0.02 ^c^	0.29 ± 0.05 ^abc^	0.26 ± 0.04 ^bc^
Isovaleric acid	3000	Acid, rancid	6 [6]	0.29 ± 0.03 ^b^	0.34 ± 0.00 ^a^	0.27 ± 0.02 ^bc^	0.22 ± 0.02 ^c^	0.27 ± 0.05 ^bc^	0.23 ± 0.02 ^c^
Hexanoic acid	420	Cheese, fatty	6 [6]	2.44 ± 0.10 ^b^	2.85 ± 0.02 ^a^	2.03 ± 0.19 ^b^	2.35 ± 0.25 ^b^	2.27 ± 0.34 ^b^	2.28 ± 0.33 ^b^
Octanoic acid	500	Rancid, cheese, fatty acid	6 [6]	0.89 ± 0.10 ^a^	0.83 ± 0.01 ^a^	0.56 ± 0.05 ^b^	0.65 ± 0.06 ^b^	0.62 ± 0.08 ^b^	0.58 ± 0.08 ^b^
Decanoic acid	1000	Fatty, rancid	6 [6]	0.29 ± 0.05 ^a^	0.20 ± 0.01 ^b^	0.16 ± 0.02 ^c^	0.16 ± 0.01 ^c^	0.15 ± 0.01 ^cd^	0.12 ± 0.03 ^d^
***Terpenes***									
*cis*-Rose oxide	0.2	Lychee	2 [6]	0.32 ± 0.01 ^a^	0.26 ± 0.01 ^a^	0.27 ± 0.05 ^a^	0.31 ± 0.02 ^a^	0.30 ± 0.01 ^a^	0.28 ± 0.05 ^a^
Geraniol	20	Citric, geranium	2 [6]	2.20 ± 0.03 ^a^	2.28 ± 0.09 ^a^	2.00 ± 0.05 ^b^	2.07 ± 0.08 ^b^	2.02 ± 0.03 ^b^	2.05 ± 0.08 ^b^
***Norisoprenoids***									
*β*-Damascenone	140	Sweet, exotic flowers, stewed apple	1,2,4 [6]	13.25 ± 0.26 ^b^	14.88 ± 1.09 ^a^	6.02 ± 0.21 ^d^	7.70 ± 0.41 ^c^	6.42 ± 0.36 ^d^	7.70 ± 0.82 ^c^
TDN	2	Kerosene, petrol	7 [58]	0.24 ± 0.01 ^c^	0.28 ± 0.04 ^c^	0.81 ± 0.08 ^a^	0.75 ± 0.07 ^ab^	0.67 ± 0.12 ^b^	0.75 ± 0.05 ^ab^
***Volatile phenols***									
Guaiacol	10	Smoky, hospital	7,8 [56]	2.45 ± 0.18 ^d^	2.61 ± 0.07 ^d^	2.98 ± 0.13 ^c^	3.43 ± 0.20 ^b^	3.36 ± 0.36 ^b^	4.31 ± 0.26 ^a^
4-Methylguaiacol	65	Smudging, toasty	8 [59]	0.13 ± 0.01 ^c^	0.14 ± 0.01 ^c^	0.20 ± 0.01 ^b^	0.21 ± 0.01 ^b^	0.22 ± 0.04 ^b^	0.34 ± 0.03 ^a^
*o*-Cresol	31	Tarry, smoke	8,9 [60]	0.11 ± 0.00 ^bc^	0.11 ± 0.01 ^bc^	0.10 ± 0.02 ^c^	0.12 ± 0.01 ^b^	0.10 ± 0.01 ^c^	0.15 ± 0.01 ^a^
*p*-Cresol	10	Tarry, smoke	8,9 [10]	4.46 ± 0.33 ^a^	4.47 ± 0.19 ^a^	3.49 ± 0.08 ^c^	3.87 ± 0.25 ^b^	3.54 ± 0.30 ^bc^	3.89 ± 0.22 ^b^
Eugenol	6	Cinnamon, clove, honey	4,5 [56]	1.01 ± 0.01 ^b^	0.84 ± 0.05 ^b^	4.73 ± 0.26 ^a^	4.74 ± 0.25 ^a^	4.78 ± 1.26 ^a^	4.93 ± 1.66 ^a^
4-Vinylguaiacol	40	Spices, curry	5 [56]	1.77 ± 0.10 ^d^	1.73 ± 0.10 ^d^	2.19 ± 0.03 ^bc^	3.23 ± 0.21 ^a^	2.01 ± 0.05 ^c^	2.23 ± 0.15 ^b^
*cis*-Isoeugenol	6	Floral	2 [61]	nd ^b^	nd ^b^	0.25 ± 0.02 ^a^	0.23 ± 0.02 ^a^	0.24 ± 0.01 ^a^	0.24 ± 0.02 ^a^
4-Vinylphenol	180	Almond shell	8 [60]	10.16 ± 0.78 ^cd^	10.85 ± 0.44 ^bc^	11.35 ± 0.39 ^bc^	18.15 ± 1.02 ^a^	9.30 ± 0.32 ^d^	11.62 ± 1.24 ^b^
***Phenolic*** ***aldehydes***									
Vanillin	200	Vanillin	4 [60]	0.35 ± 0.00 ^a^	0.36 ± 0.01 ^a^	0.18 ± 0.01 ^d^	0.20 ± 0.01 ^c^	0.16 ± 0.01^e^	0.22 ± 0.01 ^b^
***Oak lactones***									
*trans*-Whiskey lactone	67	Coconut, burn woody, vanilla	4 [61]	nd ^c^	nd ^c^	2.33 ± 0.42 ^a^	2.30 ± 0.53 ^a^	1.58 ± 0.19 ^b^	1.20 ± 0.36 ^b^
*cis*-Whiskey lactone	790	Coconut, burn woody, vanilla	4 [61]	nd ^d^	nd ^d^	0.34 ± 0.05 ^a^	0.33 ± 0.05 ^a^	0.22 ± 0.06 ^b^	0.15 ± 0.05 ^c^

^a^ Aroma series—1. fruity, 2. floral, 3. herbaceous, 4. sweet, 5. spicy, 6. fatty, 7. chemical, 8. roasty, 9. ashy. Data are mean ± standard deviation of duplicate tests. Different letters in each row represent significant differences at a significant level of 0.05. ‘nd’ represents ‘not detected’.

## 4. Conclusions

In conclusion, clone 169 wine before aging contained significantly higher concentrations of C_6_ alcohols 1-hexanol, (*E*)-3-hexen-1-ol, and (*Z*)-3-hexen-1-ol, higher alcohols 1-butanol, 1-pentanol, 3-methyl-1-pentanol, isopentanol, and 2-phenylethanol, ethyl esters ethyl butanoate, ethyl lactate, ethyl 2-hydroxy-4-methylpentanoate, isoamyl lactate, diethyl succinate, and ethyl 9-decenoate, and fatty acids hexanoic acid and butanoic acid. In contrast, acetate esters ethyl acetate and hexyl acetate, furanic compounds furfural and acetylfuran, and *cis*-rose oxide were present in significantly higher levels in clone 191 wine. After 12-month aging, some clonal differences such as C_6_ alcohols and higher alcohols remained unchanged. Meanwhile, several terpenes, norisoprenoids, volatile phenols and phenolic aldehydes showed new clonal differences, and they all had significantly higher concentrations in clone 169 wine than clone 191 wine. Moreover, the use of different oak barrels resulted in differences in the concentrations of phenolic aldehydes, volatile phenols, furanic compounds, and oak lactones in these wines. Aroma series analysis revealed that fruity and floral aromas were more prominent before aging, and decreased with duration of aging. However, the aging process led to a significant increase in the chemical, roasty, and spicy aromas of these wines. After 12-month aging, clone 169 wine exhibited higher floral and roasty aromas than clone 191 wine, while clone 191 wine had a stronger chemical aroma. Principal component analysis indicated that the aging process played a primary role in the alteration of volatile profile in these wines. Clone played a secondary role and oak barrel had tertiary contribution to the variation. This study investigated the wine-making and aging potential of clone 169 and 191, and the results showed that clone 169 was a better choice for producing high-quality aged Cabernet Sauvignon wine with intense and elegant aroma in Xinjiang region of China.

## Figures and Tables

**Table 1:** Physicochemical parameters of wines made of Cabernet Sauvignon clone 169 and 191 before aging.

Wine Samples	Reducing Sugar (g/L)	pH	Volatile Acidity (g/L)	Total Acidity (g/L)	Ethanol (%, vol)
Clone 191	3.30 ± 0.14 a	3.81 ± 0.02 a	0.47 ± 0.02 b	5.7 ± 0.10 b	14.9 ± 0.64 a
Clone 169	3.50 ± 0.21 a	3.88 ± 0.02 b	0.36 ± 0.01 a	5.3 ± 0.13 a	14.0 ± 0.14 a

Data are mean ± standard deviation of triplicate tests. Different letters between the clones represent significant difference at a significant level of 0.05.

**Table 2:** Concentration of individual volatile compounds in wine made of clone 169 and 191 before and after 12 months of aging determined by GC-MS.

Volatile	Before Aging (μg/L)		Y169/Y191 ^a^	12 Month Aging (μg/L)				*p* Value ^b^		
	Clone 191	Clone 169		Clone 191 (Wolin)	Clone 169 (Wolin)	Clone 191 (Bordeaux)	Clone 169 (Bordeaux)	Clone	Barrel	Clone × Barrel
*C_6_ alcohols*										
1-Hexanol (mg/L)	2.34 ± 0.01 ^b*^	2.54 ± 0.04 ^a^	**1.09** **	2.06 ± 0.15 ^c^	2.26 ± 0.09 ^b^	2.07 ± 0.04 ^c^	2.29 ± 0.07 ^b^	**<0.01**	0.37	0.73
(*E*)-3-Hexen-1-ol	50.13 ± 1.17 ^b^	58.67 ± 0.18 ^a^	**1.17**	43.01 ± 4.76 ^cd^	45.90 ± 0.77 ^c^	41.04 ± 2.26 ^d^	47.05 ± 2.26 ^bc^	**0.01**	0.69	0.18
(*Z*)-3-Hexen-1-ol	66.27 ± 1.87 ^b^	73.47 ± 1.44 ^a^	**1.11**	58.50 ± 3.96 ^c^	61.32 ± 4.10 ^bc^	60.10 ± 5.73 ^bc^	57.60 ± 5.55 ^c^	0.16	**<0.01**	0.64
(*E*)-2-Hexen-1-ol	34.65 ± 2.55 ^a^	31.59 ± 2.10 ^a^	0.91	17.53 ± 2.14 ^b^	17.37 ± 0.04 ^b^	17.88 ± 1.14 ^b^	15.17 ± 1.17 ^b^	0.98	0.07	0.64
(*Z*)-2-Hexen-1-ol	27.85 ± 1.37 ^b^	21.13 ± 1.44 ^d^	**0.76**	30.84 ± 2.03 ^a^	27.46 ± 0.87 ^b^	29.00 ± 2.08 ^ab^	24.91 ± 1.01 ^c^	**<0.01**	**0.02**	0.86
*Higher alcohols*										
1-Butanol (mg/L)	1.77 ± 0.01 ^d^	2.57 ± 0.04 ^a^	**1.45**	1.86 ± 0.05 ^c^	2.40 ± 0.01 ^b^	1.87 ± 0.01 ^c^	2.44 ± 0.06 ^b^	**<0.01**	0.49	0.53
Isobutanol (mg/L)	39.61 ± 0.36 ^c^	41.74 ± 0.12 ^b^	**1.05**	44.14 ± 1.06 ^a^	41.30 ± 0.57 ^b^	44.06 ± 0.43 ^a^	42.47 ± 1.63 ^b^	0.41	0.51	0.45
1-Pentanol	93.50 ± 4.67 ^c^	136.85 ± 0.74 ^b^	**1.46**	128.71 ± 10.30 ^b^	155.35 ± 10.32 ^a^	123.76 ± 5.38 ^b^	156.22 ± 7.37 ^a^	**<0.01**	0.09	0.06
Isopentanol (mg/L)	206.14 ± 1.17 ^bc^	233.48 ± 0.58 ^a^	**1.13**	201.10 ± 6.54 ^c^	209.57 ± 2.19 ^b^	201.31 ± 1.33 ^c^	210.80 ± 3.14 ^b^	**<0.01**	0.65	0.75
3-Methyl-1-pentanol	123.61 ± 0.39 ^c^	159.57 ± 0.50 ^a^	**1.29**	105.26 ± 1.62 ^d^	129.28 ± 2.10 ^b^	105.51 ± 0.84 ^d^	128.49 ± 1.30 ^b^	**<0.01**	0.44	0.59
4-Methyl-1-pentanol	55.88 ± 0.56 ^b^	63.53 ± 2.98 ^a^	1.14	46.30 ± 6.73 ^cd^	50.21 ± 4.36 ^c^	45.54 ± 2.35 ^d^	50.07 ± 2.68 ^c^	**<0.01**	0.85	0.71
1-Octanol	8.19 ± 0.24 ^a^	8.33 ± 0.93 ^a^	1.02	2.69 ± 0.03 ^b^	2.61 ± 0.26 ^b^	2.49 ± 0.05 ^c^	2.56 ± 0.42 ^bc^	**0.02**	0.22	0.38
3-Octanol	0.85 ± 0.01 ^a^	0.90 ± 0.05 ^a^	1.05	0.68 ± 0.06 ^b^	0.82 ± 0.04 ^a^	0.70 ± 0.04 ^b^	0.71 ± 0.02 ^b^	**0.04**	0.14	0.07
1-Octen-3-ol	7.35 ± 0.11 ^b^	6.11 ± 0.16 ^c^	**0.83**	8.81 ± 0.54 ^a^	7.61 ± 0.31 ^b^	8.07 ± 0.54 ^b^	7.89 ± 0.24 ^b^	0.1	0.88	0.27
2-Ethyl-1-hexanol	10.93 ± 1.32 ^a^	9.36 ± 0.21 ^a^	0.86	5.42 ± 0.37 ^c^	4.92 ± 0.36 ^c^	7.33 ± 0.65 ^b^	6.00 ± 0.68 ^bc^	0.49	0.92	0.93
2-Nonanol	0.70 ± 0.08 ^b^	0.70 ± 0.03 ^b^	1.01	0.97 ± 0.07 ^a^	1.05 ± 0.08 ^a^	0.98 ± 0.05 ^a^	0.96 ± 0.06 ^a^	0.33	0.22	0.23
1-Decanol	2.48 ± 0.05 ^a^	2.38 ± 0.22 ^a^	0.96	1.50 ± 0.03 ^b^	1.66 ± 0.06 ^b^	1.54 ± 0.02 ^b^	1.64 ± 0.03 ^b^	**<0.01**	0.28	**0.02**
1-Dodecanol	3.23 ± 0.11 ^c^	3.18 ± 0.04 ^c^	0.98	5.99 ± 0.58 ^a^	6.61 ± 0.55 ^a^	6.34 ± 0.82 ^a^	4.70 ± 0.72 ^b^	0.53	0.15	0.31
(*Z*)-6-Nonen-1-ol	5.91 ± 0.89 ^a^	3.62 ± 0.30 ^b^	0.61	1.64 ± 0.05 ^c^	1.69 ± 0.12 ^c^	1.72 ± 0.17 ^c^	1.80 ± 0.05 ^c^	0.37	0.18	0.81
Benzyl alcohol	775.25 ± 14.49 ^bc^	807.57 ± 0.52 ^ab^	1.04	641.25 ± 45.49 ^c^	862.46 ± 84.30 ^ab^	877.46 ± 120.87 ^ab^	940.59 ± 64.14 ^a^	**<0.01**	**0.02**	25.92
2-Phenylethanol (mg/L)	32.39 ± 1.30 ^b^	38.56 ± 0.36 ^a^	**1.19**	18.89 ± 1.37 ^d^	24.87 ± 2.96 ^c^	24.12 ± 1.28 ^cd^	27.40 ± 5.16 ^bc^	0.1	0.15	0.57
*Acetate esters*										
Ethyl acetate (mg/L)	64.58 ± 0.64 ^d^	51.18 ± 0.98^e^	**0.79**	124.56 ± 4.12 ^b^	97.85 ± 1.34 ^c^	129.38 ± 4.05 ^a^	95.56 ± 1.13 ^c^	**<0.01**	0.23	**0.02**
Isoamyl acetate	447.38 ± 4.30 ^a^	461.89 ± 23.65 ^a^	1.03	366.51 ± 16.00 ^c^	351.62 ± 12.16 ^cd^	404.63 ± 17.32 ^b^	327.14 ± 22.17 ^d^	**0.01**	0.5	**0.03**
Hexyl acetate	2.57 ± 0.01 ^a^	2.06 ± 0.11 ^b^	**0.80**	2.11 ± 0.13 ^b^	1.57 ± 0.14 ^c^	2.50 ± 0.13 ^a^	1.92 ± 0.16 ^b^	**<0.01**	**0.01**	0.83
2-Ethyl-1-hexyl acetate	9.41 ± 0.20 ^a^	8.66 ± 0.76 ^a^	0.92	1.15 ± 0.08 ^b^	0.92 ± 0.05 ^b^	1.44 ± 0.07 ^b^	0.90 ± 0.20 ^b^	**0.01**	0.34	0.26
Phenethyl acetate	13.30 ± 0.05 ^a^	12.45 ± 0.47 ^b^	0.94	9.21 ± 0.36 ^d^	9.31 ± 0.35 ^d^	10.02 ± 0.48 ^c^	9.50 ± 0.22 ^cd^	0.15	**0.01**	0.05
*Ethyl esters*										
Ethyl butanoate	216.24 ± 2.23 ^b^	237.81 ± 6.26 ^a^	**1.10**	206.30 ± 8.48 ^c^	219.98 ± 3.88 ^b^	215.66 ± 10.23 ^b^	216.84 ± 4.32 ^b^	**0.03**	0.22	**0.04**
Ethyl hexanoate	411.35 ± 4.45 ^a^	392.91 ± 24.72 ^a^	0.96	319.30 ± 19.68 ^b^	331.86 ± 14.81 ^b^	335.59 ± 17.95 ^b^	335.97 ± 6.07 ^b^	**0.03**	**<0.01**	**0.04**
Ethyl 2-hexenoate	8.72 ± 0.03 ^a^	6.72 ± 0.48 ^b^	**0.77**	6.13 ± 0.22 ^c^	5.55 ± 0.25 ^d^	6.43 ± 0.32 ^bc^	5.61 ± 0.06 ^d^	**<0.01**	**0.01**	**0.04**
Ethyl heptanoate	1.39 ± 0.01 ^ab^	1.42 ± 0.16 ^ab^	1.03	1.26 ± 0.11 ^c^	1.29 ± 0.10 ^bc^	1.48 ± 0.11 ^a^	1.50 ± 0.08 ^a^	0.3	**<0.01**	0.51
Ethyl lactate (mg/L)	21.97 ± 0.09 ^d^	53.02 ± 1.56 ^c^	**2.41**	197.22 ± 14.55 ^a^	219.69 ± 10.13 ^a^	158.48 ± 0.84 ^b^	218.14 ± 21.52 ^a^	**0.04**	0.52	0.26
Ethyl octanoate	556.83 ± 1.63 ^a^	600.48 ± 42.72 ^a^	1.08	358.76 ± 27.73 ^b^	390.53 ± 19.68 ^b^	387.09 ± 14.76 ^b^	377.98 ± 6.14 ^b^	0.27	0.43	0.08
Ethyl nonanoate	2.81 ± 0.01 ^a^	2.81 ± 0.21 ^a^	1.00	1.66 ± 0.10 ^b^	1.60 ± 0.06 ^b^	1.76 ± 0.04 ^b^	1.66 ± 0.03 ^b^	0.1	0.11	0.62
Ethyl decanoate	336.35 ± 0.40 ^a^	328.40 ± 25.46 ^a^	0.98	167.63 ± 5.20 ^b^	163.57 ± 4.32 ^b^	166.26 ± 2.19 ^b^	159.43 ± 1.44 ^b^	**0.03**	0.17	0.45
Ethyl 2-hydroxy-4-methylpentanoate	7.19 ± 0.16 ^d^	9.09 ± 0.20 ^d^	**1.26**	29.52 ± 2.92 ^a^	27.41 ± 1.82 ^ab^	25.88 ± 0.60 ^bc^	24.65 ± 1.89 ^c^	0.13	0.14	0.8
Ethyl furoate	20.84 ± 1.44 ^d^	26.18 ± 5.18 ^cd^	1.26	31.76 ± 1.03 ^bc^	34.72 ± 1.73 ^ab^	33.75 ± 1.05 ^ab^	39.63 ± 3.03 ^a^	**0.03**	0.06	0.33
Ethyl benzoate	0.59 ± 0.01 ^b^	0.65 ± 0.01 ^a^	1.10	0.40 ± 0.04 ^d^	0.53 ± 0.03 ^c^	0.41 ± 0.03 ^d^	0.52 ± 0.01 ^c^	**<0.01**	0.44	0.84
Ethyl undecanoate	0.18 ± 0.00 ^a^	0.14 ± 0.01 ^b^	0.78	0.04 ± 0.00 ^c^	0.04 ± 0.01 ^c^	0.02 ± 0.01 ^d^	0.03 ± 0.00 ^cd^	0.47	**0.04**	0.25
Ethyl 9-decenoate	1.67 ± 0.06 ^b^	2.65 ± 0.31 ^a^	**1.59**	0.48 ± 0.10 ^c^	0.56 ± 0.03 ^c^	0.39 ± 0.07 ^c^	0.50 ± 0.04 ^c^	**0.03**	0.06	0.43
Ethyl phenylacetate	1.67 ± 0.04 ^b^	1.50 ± 0.04 ^c^	0.90	1.85 ± 0.04 ^a^	1.81 ± 0.07 ^a^	1.87 ± 0.10 ^a^	1.87 ± 0.04 ^a^	0.69	0.35	0.6
Ethyl dodecanoate	32.79 ± 1.27 ^a^	31.60 ± 4.02 ^a^	0.96	16.33 ± 0.29 ^b^	16.97 ± 0.27 ^b^	16.23 ± 0.34 ^b^	16.22 ± 0.29 ^b^	0.14	0.07	0.13
Ethyl dihydrocinnamate	0.57 ± 0.03 ^ab^	0.82 ± 0.13 ^a^	1.43	0.28 ± 0.02 ^c^	0.39 ± 0.07 ^bc^	0.41 ± 0.01 ^b^	0.37 ± 0.03 ^bc^	0.18	0.74	0.57
Ethyl myristate	0.91 ± 0.08 ^b^	1.07 ± 0.04 ^a^	1.18	0.53 ± 0.01 ^c^	0.61 ± 0.05 ^c^	0.49 ± 0.05 ^cd^	0.36 ± 0.02 ^d^	1	**<0.01**	0.12
Ethyl palmitate	0.94 ± 0.04 ^a^	0.94 ± 0.31 ^a^	1.00	0.63 ± 0.04 ^bc^	0.81 ± 0.08 ^ab^	0.68 ± 0.11 ^bc^	0.58 ± 0.03 ^c^	0.4	0.16	0.28
Diethyl succinate (mg/L)	2.17 ± 0.03 ^b^	3.02 ± 0.06 ^b^	**1.40**	13.43 ± 0.89 ^a^	13.83 ± 1.14 ^a^	13.13 ± 1.01 ^a^	15.26 ± 1.34 ^a^	0.47	0.4	0.82
*Other esters*										
Methyl octanoate	1.96 ± 0.03 ^a^	2.05 ± 0.26 ^a^	1.04	1.10 ± 0.09 ^b^	1.24 ± 0.09 ^b^	1.30 ± 0.08 ^b^	1.25 ± 0.03 ^b^	0.16	**0.03**	**0.04**
Isoamyl hexanoate	5.25 ± 0.01 ^b^	5.50 ± 0.16 ^a^	1.05	4.91 ± 0.06 ^c^	5.02 ± 0.07 ^c^	4.87 ± 0.04 ^c^	4.96 ± 0.01 ^c^	**<0.01**	0.06	0.74
Isobutyl hexanoate	4.73 ± 1.03 ^ab^	5.69 ± 0.46 ^a^	1.20	4.17 ± 0.29 ^b^	4.45 ± 0.33 ^b^	4.40 ± 0.37 ^b^	4.26 ± 0.22 ^b^	0.49	0.85	0.09
Methyl salicylate	3.04 ± 0.12 ^a^	2.42 ± 0.05 ^c^	**0.80**	2.79 ± 0.15 ^ab^	2.79 ± 0.10 ^ab^	2.59 ± 0.06 ^bc^	2.52 ± 0.21 ^bc^	0.94	0.21	0.9
Propyl octanoate	2.27 ± 0.24 ^a^	2.54 ± 0.33 ^a^	1.12	0.97 ± 0.08 ^b^	1.07 ± 0.10 ^b^	0.87 ± 0.08 ^b^	1.05 ± 0.09 ^b^	**0.04**	0.48	0.72
Isoamyl octanoate	36.30 ± 0.66 ^b^	42.83 ± 5.19 ^a^	1.18	16.34 ± 0.74 ^c^	17.28 ± 0.67 ^c^	15.79 ± 0.38 ^c^	16.03 ± 0.31 ^c^	**0.04**	**0.01**	0.17
Isobutyl octanoate	5.70 ± 0.10 ^b^	6.96 ± 0.68 ^a^	1.22	2.19 ± 0.12 ^c^	2.17 ± 0.21 ^c^	1.91 ± 0.15 ^c^	1.92 ± 0.05 ^c^	0.58	0.09	0.63
Isoamyl lactate	39.19 ± 5.75 ^c^	64.35 ± 3.56 ^c^	**1.64**	237.46 ± 21.12 ^b^	325.03 ± 29.01 ^a^	192.76 ± 6.96 ^b^	314.23 ± 27.36 ^a^	**<0.01**	0.17	0.46
Methyl decanoate	4.26 ± 0.01 ^a^	3.98 ± 0.51 ^a^	0.93	0.92 ± 0.07 ^b^	0.82 ± 0.06 ^b^	0.93 ± 0.03 ^b^	0.83 ± 0.02 ^b^	**0.02**	0.7	0.97
Methyl laurate	0.18 ± 0.03 ^a^	0.15 ± 0.04 ^a^	0.81	0.06 ± 0.01 ^b^	0.06 ± 0.01 ^b^	0.06 ± 0.01 ^b^	0.04 ± 0.01 ^b^	**0.04**	0.43	0.43
Isopentyl decanoate	18.97 ± 1.18 ^a^	18.06 ± 5.32 ^a^	0.95	3.39 ± 0.33 ^b^	3.56 ± 0.39 ^b^	2.73 ± 0.19 ^b^	2.81 ± 0.34 ^b^	0.35	**0.01**	0.62
*Fatty acids*										
Propanoic acid	731.48 ± 105.59 ^c^	947.47 ± 19.73 ^bc^	1.30	1010.84 ± 96.32 ^b^	951.40 ± 95.11 ^bc^	1051.44 ± 87.44 ^b^	1298.21 ± 126.66 ^a^	0.51	**0.03**	0.2
Isobutyric acid	795.43 ± 17.93 ^a^	688.84 ± 111.48 ^ab^	0.87	704.41 ± 63.00 ^ab^	561.95 ± 36.01 ^b^	617.54 ± 31.08 ^b^	632.65 ± 81.36 ^b^	0.09	0.92	0.53
Isovaleric acid	858.84 ± 99.48 ^b^	1023.07 ± 7.99 ^a^	1.19	798.66 ± 74.96 ^bc^	686.42 ± 67.29 ^c^	731.94 ± 30.35 ^bc^	699.42 ± 64.56 ^c^	**0.05**	0.69	0.74
Hexanoic acid	1022.90 ± 42.01 ^ab^	1196.13 ± 6.22 ^a^	**1.17**	851.48 ± 78.70 ^b^	941.24 ± 66.55 ^b^	1011.46 ± 100.62 ^ab^	957.15 ± 138.99 ^b^	0.34	0.61	0.37
Octanoic acid	445.52 ± 52.02 ^a^	415.31 ± 4.53 ^a^	0.93	279.56 ± 25.76 ^b^	324.01 ± 32.14 ^b^	327.25 ± 24.14 ^b^	271.18 ± 21.12 ^b^	0.56	0.94	0.19
Butanoic acid	690.70 ± 43.13 ^c^	1059.52 ± 13.97 ^a^	**1.53**	766.02 ± 73.38 ^bc^	781.65 ± 73.55 ^bc^	726.46 ± 28.13 ^c^	895.94 ± 88.95 ^b^	0.64	0.39	0.81
Decanoic acid	290.17 ± 47.89 ^a^	198.78 ± 5.81 ^b^	0.69	158.22 ± 2.28 ^bc^	160.81 ± 10.56 ^bc^	146.31 ± 9.71 ^bc^	126.09 ± 20.13 ^c^	0.12	**0.01**	0.06
*Terpenes*										
*cis*-Rose oxide (ng/L)	63.39 ± 1.22 ^a^	52.62 ± 2.44 ^b^	**0.83**	57.66 ± 5.01 ^ab^	61.23 ± 5.06 ^ab^	59.56 ± 2.56 ^ab^	60.17 ± 8.01 ^ab^	0.42	0.78	0.11
Linalool	1.00 ± 0.04 ^c^	1.05 ± 0.04 ^c^	1.06	1.15 ± 0.06 ^b^	1.24 ± 0.03 ^a^	1.15 ± 0.05 ^b^	1.23 ± 0.03 ^ab^	**0.02**	0.7	0.7
Citronellyl acetate (ng/L)	566.46 ± 16.76 ^c^	548.90 ± 8.95 ^c^	0.97	1181.30 ± 78.68 ^bc^	1259.14 ± 55.20 ^bc^	1835.88 ± 522.79 ^ab^	2016.56 ± 162.96 ^a^	**0.01**	**<0.01**	0.07
Citronellol	4.56 ± 0.16 ^a^	5.00 ± 0.74 ^a^	1.10	1.38 ± 0.05 ^b^	1.87 ± 0.11 ^b^	1.39 ± 0.02 ^b^	1.92 ± 0.19 ^b^	**0.02**	0.87	0.79
Nerol	35.29 ± 3.13 ^a^	33.31 ± 0.69 ^a^	0.94	33.92 ± 0.73 ^a^	33.33 ± 0.39 ^a^	33.65 ± 0.45 ^a^	33.11 ± 0.45 ^a^	**0.05**	0.28	0.89
*α*-Terpineol (ng/L)	645.41 ± 17.09 ^b^	639.05 ± 6.21 ^b^	0.99	784.05 ± 52.98 ^a^	870.56 ± 33.39 ^a^	809.56 ± 46.15 ^a^	859.30 ± 57.95 ^a^	0.1	0.83	0.59
Methyl geranate	0.35 ± 0.04 ^a^	0.39 ± 0.01 ^a^	1.10	0.07 ± 0.01 ^b^	0.08 ± 0.01 ^b^	0.07 ± 0.01 ^b^	0.07 ± 0.01 ^b^	0.43	0.33	0.54
Geranylacetone	589.10 ± 9.23 ^b^	589.01 ± 192.79 ^b^	1.00	860.48 ± 48.94 ^a^	897.26 ± 47.11 ^a^	904.25 ± 62.87 ^a^	916.84 ± 57.00 ^a^	0.55	0.45	0.77
Geraniol	43.96 ± 0.60 ^ab^	45.65 ± 1.85 ^a^	1.04	40.10 ± 0.99 ^c^	41.50 ± 1.67 ^bc^	40.35 ± 0.55 ^c^	41.23 ± 1.68 ^c^	0.2	0.88	0.63
*Norisoprenoids*										
*β*-Damascenone (ng/L)	1854.80 ± 36.71 ^b^	2083.75 ± 153.17 ^a^	1.12	843.12 ± 28.84 ^d^	1078.59 ± 57.50 ^c^	899.28 ± 49.80 ^d^	1129.11 ± 64.70 ^c^	**0.01**	0.55	0.55
Riesling acetal	0.73 ± 0.04 ^a^	0.65 ± 0.04 ^b^	0.89	0.55 ± 0.03 ^c^	0.57 ± 0.03 ^c^	0.50 ± 0.03 ^c^	0.57 ± 0.02 ^c^	**0.04**	0.26	0.26
Vitispirane A	0.58 ± 0.03 ^d^	0.65 ± 0.08 ^d^	1.11	2.21 ± 0.14 ^b^	2.43 ± 0.11 ^a^	1.98 ± 0.08 ^c^	2.24 ± 0.07 ^b^	**0.01**	**0.02**	0.7
Vitispirane B	0.47 ± 0.03 ^d^	0.52 ± 0.02 ^d^	1.10	1.33 ± 0.10 ^b^	1.48 ± 0.07 ^a^	1.21 ± 0.04 ^c^	1.43 ± 0.03 ^a^	**<0.01**	**0.04**	0.29
TDN	0.48 ± 0.03 ^b^	0.56 ± 0.08 ^b^	1.17	1.55 ± 0.12 ^a^	1.50 ± 0.15 ^a^	1.25 ± 0.20 ^a^	1.49 ± 0.10 ^a^	0.93	0.31	0.35
*Volatile phenols*										
Guaiacol	23.32 ± 1.66 ^d^	24.76 ± 0.65 ^d^	1.06	28.34 ± 0.12 ^c^	32.56 ± 0.64 ^b^	31.95 ± 4.24 ^b^	40.92 ± 0.52 ^a^	**0.01**	**0.02**	0.2
4-Methylguaiacol	8.74 ± 0.73 ^c^	9.19 ± 0.01 ^c^	1.05	12.73 ± 0.41 ^b^	13.43 ± 0.33 ^b^	14.15 ± 3.57 ^b^	22.32 ± 2.46 ^a^	**0.05**	**0.03**	0.07
*o*-Cresol	3.53 ± 0.13 ^bc^	3.54 ± 0.27 ^bc^	1.00	3.17 ± 0.58 ^c^	3.86 ± 0.51 ^b^	3.21 ± 0.05 ^c^	4.64 ± 0.31 ^a^	**0.02**	0.2	0.34
Phenol	31.66 ± 1.38 ^bc^	31.11 ± 0.12 ^c^	0.98	30.24 ± 0.34 ^c^	34.43 ± 1.02 ^ab^	29.42 ± 0.30 ^c^	37.45 ± 2.63 ^a^	**<0.01**	0.34	0.13
4-Ethylguaiacol	1.44 ± 0.04 ^bc^	1.36 ± 0.09 ^c^	0.94	1.69 ± 0.05 ^b^	1.76 ± 0.19 ^b^	1.78 ± 0.38 ^b^	2.74 ± 0.26 ^a^	**0.05**	**0.04**	0.07
*p*-Cresol	44.58 ± 3.30 ^a^	44.71 ± 1.95 ^a^	1.00	34.94 ± 0.93 ^b^	38.71 ± 0.31 ^b^	35.39 ± 2.05 ^b^	38.87 ± 2.08 ^b^	**0.03**	0.8	0.9
*m*-Cresol	3.65 ± 0.36 ^b^	3.20 ± 0.04 ^bc^	0.88	2.68 ± 0.22 ^c^	3.27 ± 0.30 ^bc^	3.21 ± 0.24 ^bc^	4.94 ± 0.16 ^a^	**<0.01**	**<0.01**	**0.03**
4-Propylguaiacol	1.02 ± 0.04 ^c^	0.90 ± 0.03 ^c^	0.89	1.00 ± 0.18 ^c^	1.46 ± 0.23 ^ab^	1.24 ± 0.06 ^bc^	1.76 ± 0.29 ^a^	**0.03**	0.14	0.84
Eugenol	6.05 ± 0.03 ^b^	5.06 ± 0.32 ^b^	0.84	28.40 ± 1.86 ^a^	28.46 ± 0.95 ^a^	28.70 ± 9.19 ^a^	29.60 ± 12.12 ^a^	0.93	0.9	0.94
4-Ethylphenol	14.34 ± 0.08 ^b^	16.99 ± 0.20 ^a^	1.19	14.57 ± 0.16 ^b^	15.99 ± 0.64 ^a^	12.82 ± 0.10 ^c^	14.68 ± 1.22 ^b^	**0.03**	**0.04**	0.68
4-Vinylguaiacol	70.70 ± 3.98 ^d^	69.39 ± 3.95 ^d^	0.98	87.79 ± 0.23 ^bc^	129.23 ± 3.39 ^a^	80.44 ± 0.56 ^c^	89.14 ± 4.30 ^b^	**<0.01**	**<0.01**	**<0.01**
*cis*-Isoeugenol	nd ^b^	nd ^b^		1.48 ± 0.12 ^a^	1.36 ± 0.16 ^a^	1.42 ± 0.08 ^a^	1.47 ± 0.12 ^a^	0.7	0.78	0.39
*trans*-Isoeugenol	1.90 ± 0.04 ^c^	1.69 ± 0.01^e^	0.89	2.48 ± 0.02 ^a^	1.82 ± 0.07 ^cd^	2.21 ± 0.00 ^b^	1.77 ± 0.07 ^de^	**<0.01**	**0.01**	**0.04**
4-Vinylphenol (mg/L)	1.83 ± 0.14 ^bc^	1.95 ± 0.08 ^bc^	1.07	2.04 ± 0.06 ^b^	3.27 ± 0.09 ^a^	1.67 ± 0.01 ^c^	2.09 ± 0.23 ^b^	**<0.01**	**<0.01**	**0.01**
Syringol	205.71 ± 9.27 ^c^	196.59 ± 5.36 ^c^	0.96	330.17 ± 9.93 ^b^	342.41 ± 12.67 ^ab^	347.83 ± 22.01 ^ab^	373.88 ± 14.15 ^a^	0.15	0.09	0.56
*Phenolic aldehydes*										
Vanillin	70.15 ± 0.06 ^a^	72.26 ± 1.39 ^a^	1.03	35.92 ± 1.03 ^d^	40.41 ± 1.42 ^c^	31.95 ± 1.28^e^	44.46 ± 0.07 ^b^	**<0.01**	0.72	**<0.01**
Acetovanilone	60.89 ± 2.33 ^b^	64.23 ± 6.41 ^b^	1.06	56.73 ± 1.15 ^b^	64.79 ± 1.21 ^b^	64.60 ± 10.33 ^b^	97.64 ± 7.46 ^a^	**<0.01**	**<0.01**	**<0.01**
Syringaldehyde	6.62 ± 0.27 ^d^	6.28 ± 0.39 ^d^	0.95	19.43 ± 2.46 ^c^	44.45 ± 11.55 ^b^	56.61 ± 8.89 ^a^	52.18 ± 2.04 ^ab^	**0.01**	**<0.01**	**<0.01**
Acetosyringone	21.34 ± 1.15 ^c^	21.38 ± 1.32 ^c^	1.00	35.43 ± 0.92 ^bc^	40.24 ± 2.39 ^b^	45.72 ± 11.78 ^b^	90.72 ± 7.16 ^a^	**<0.01**	**<0.01**	**<0.01**
*Furanic compounds*										
Furfural	5.10 ± 0.15 ^c^	0.91 ± 0.01 ^d^	**0.18**	36.57 ± 0.71 ^b^	25.90 ± 13.26 ^b^	298.14 ± 36.68 ^a^	300.74 ± 44.77 ^a^	0.96	**<0.01**	0.93
5-Methyl furfural	nd ^b^	nd ^b^		nd ^b^	nd ^b^	121.05 ± 0.17 ^a^	71.11 ± 14.81 ^a^	0.85	**<0.01**	0.85
Acetylfuran	6.27 ± 0.19 ^c^	4.65 ± 0.16 ^d^	**0.74**	18.19 ± 0.53 ^bc^	20.00 ± 4.99 ^bc^	34.64 ± 14.52 ^b^	59.73 ± 5.28 ^a^	**<0.01**	**<0.01**	**<0.01**
Maltol	117.68 ± 7.71 ^c^	110.93 ± 5.23 ^c^	0.94	216.55 ± 0.28 ^b^	220.01 ± 14.34 ^b^	234.36 ± 12.62 ^b^	331.63 ± 6.36 ^a^	**<0.01**	**<0.01**	**<0.01**
Cyclotene	1.04 ± 0.03 ^c^	1.09 ± 0.04 ^c^	1.05	4.41 ± 0.07 ^b^	5.25 ± 0.64 ^a^	4.66 ± 0.27 ^ab^	4.06 ± 0.19 ^b^	0.29	**0.05**	**<0.01**
*Oak lactones*										
*trans*-Whiskey lactone	nd ^c^	nd ^c^		155.81 ± 34.43 ^a^	154.35 ± 41.43 ^a^	106.04 ± 14.88 ^b^	80.29 ± 29.08 ^b^	0.32	**<0.01**	0.36
*cis*-Whiskey lactone	nd ^d^	nd ^d^		265.85 ± 52.08 ^a^	263.26 ± 46.32 ^a^	177.55 ± 61.37 ^b^	116.81 ± 46.35 ^c^	0.16	**<0.01**	0.2
*Carbonyl compounds*										
Acetoin (mg/L)	1.38 ± 0.03 ^d^	2.47 ± 0.18 ^c^	**1.79**	5.21 ± 0.18 ^a^	5.12 ± 0.36 ^a^	3.82 ± 0.38 ^b^	3.91 ± 0.17 ^b^	0.14	**<0.01**	0.62
Benzaldehyde	18.49 ± 0.85 ^bc^	18.92 ± 0.04 ^abc^	1.02	18.59 ± 0.37 ^bc^	19.85 ± 0.15 ^ab^	17.95 ± 1.78 ^c^	20.00 ± 0.21 ^a^	**0.02**	0.63	0.45
Benzeneacetaldehyde	83.52 ± 4.89 ^ab^	86.90 ± 3.64 ^a^	1.04	74.37 ± 3.43 ^b^	81.16 ± 5.58 ^ab^	82.17 ± 3.97 ^ab^	85.48 ± 5.38 ^a^	0.15	0.1	0.57
Decanal	2.58 ± 0.05 ^a^	2.48 ± 0.18 ^a^	0.96	1.78 ± 0.07 ^b^	1.51 ± 0.07 ^b^	1.79 ± 0.15 ^b^	1.72 ± 0.22 ^b^	0.97	0.3	0.31
*Benzenes*										
Styrene	4.20 ± 0.21 ^b^	4.44 ± 0.01 ^a^	1.06	3.41 ± 0.04 ^c^	3.46 ± 0.07 ^c^	3.58 ± 0.04 ^c^	3.58 ± 0.09 ^c^	0.52	**0.01**	0.4
Naphthalene	1.06 ± 0.02 ^d^	1.04 ± 0.01 ^d^	0.98	1.45 ± 0.01 ^c^	1.48 ± 0.03 ^bc^	1.54 ± 0.02 ^ab^	1.58 ± 0.04 ^a^	0.19	**0.02**	0.89
1-Methylnaphthalene	0.05 ± 0.01 ^c^	0.03 ± 0.01 ^c^	0.67	0.26 ± 0.01 ^b^	0.30 ± 0.02 ^ab^	0.32 ± 0.02 ^ab^	0.35 ± 0.04 ^a^	0.2	0.09	0.87
*others*										
Methionol	703.42 ± 16.31 ^b^	953.59 ± 50.82 ^a^	**1.36**	448.91 ± 24.81 ^c^	516.17 ± 39.67 ^c^	475.17 ± 75.22 ^c^	605.09 ± 78.41 ^bc^	0.15	0.53	0.51
3-Isobutyl-2-methoxypyrazine	trace	trace		trace	trace	trace	trace			

^a^ The concentration ratio of the clone 169 wine to the clone 191 wine before aging. ^b^ *p* values of a two-way ANOVA for the effects of clone, barrel, and their interactions (bold values indicate *p* ≤ 0.05). * Different letters in each row indicate significant difference at *p* ≤ 0.05 using one-way ANOVA. ** Bold values indicate significant difference (*p* ≤ 0.05) between wines made of clone 169 and 191 before aging using *t*-test. ‘nd’ represents ‘not detected’. ‘trace’ means volatile cannot be quantified.

## Data Availability

All data generated or analyzed during this study are included in this article.

## Supplementary Materials

The following are available online at https://www.mdpi.com/article/10.3390/foods11010074/s1, Table S1: Volatile compounds identified in wine made of clone 169 and 191.
