# Supplementary material for: Characterization and Evolution of Volatile Compounds of Cabernet Sauvignon Wines from Two Different Clones during Oak Barrel Aging
Source: Foods. 2021 Dec 29;11(1):74. doi: 10.3390/foods11010074 (PMC8750660; doi:10.3390/foods11010074)
Supplement: Supplementary file 1 [file foods-11-00074-s001.zip › foods-1515313-supplementary.pdf]

Table S1 Volatile compounds identified in wine made of clone 169 and 191.

NO.	Compounds	RI[a]	ID[b]	Purity	Manufacturer
	C_6 alcohols				
A1	1-Hexanol	1353.00	A	0.98	Sigma–Aldrich
A2	(*E*)-3-Hexen-1-ol	1365.00	A	0.97	Sigma–Aldrich
A3	(*Z*)-3-Hexen-1-ol	1387.00	A	0.98	Sigma–Aldrich
A4	(*E*)-2-Hexen-1-ol	1409.00	A	0.96	Sigma–Aldrich
A5	(*Z*)-2-Hexen-1-ol	1417.00	A	0.95	Sigma–Aldrich
	Higher alcohols				
B1	1-Butanol	1158.00	A	0.99	Sigma–Aldrich
B2	Isobutanol	1111.00	A	0.99	Sigma–Aldric
B3	1-Pentanol	1256.00	A	0.99	Sigma–Aldrich
B4	Isopentanol	1220.00	A	0.99	Sigma–Aldrich
B5	3-Methyl-1-pentanol	1328.00	B		
B6	4-Methyl-1-pentanol	1316.00	A	0.97	Sigma–Aldrich
B7	1-Octanol	1557.00	A	0.99	Sigma–Aldrich
B8	3-Octanol	1392.00	B		
B9	1-Octen-3-ol	1451.00	A	0.98	Sigma–Aldrich
B10	2-Ethyl-1-hexanol	1490.00	A	0.99	Sigma–Aldrich
B11	2-Nonanol	1517.00	A	0.99	Sigma–Aldrich
B12	1-Decanol	1765.00	A	0.99	Sigma–Aldrich
B13	1-Dodecanol	1974.00	A	0.98	Sigma–Aldrich
B14	(*Z*)-6-Nonen-1-ol	1720.00	B		
B15	Benzyl alcohol	1892.00	A	0.98	Sigma–Aldrich
B16	2-Phenylethanol	1928.00	A	0.99	Sigma–Aldrich
	Acetate esters				
C1	Ethyl acetate	858.00	B		
C2	Isoamyl acetate	1122.00	A	0.95	Sigma–Aldrich
C3	Hexyl acetate	1273.00	A	0.99	Sigma–Aldrich
C4	2-Ethyl-1-hexyl acetate	1420.00	B		
C5	Phenethyl acetate	1830.00	A	0.99	Sigma–Aldrich
	Ethyl esters				
D1	Ethyl butanoate	1047.00	A	0.99	Sigma–Aldrich
D2	Ethyl hexanoate	1232.00	A	0.99	Sigma–Aldrich
D3	Ethyl 2-hexenoate	1346.00	B		
D4	Ethyl heptanoate	1334.00	B		
D5	Ethyl lactate	1350.00	A	0.98	Sigma–Aldrich
D6	Ethyl octanoate	1437.00	A	0.99	Sigma–Aldrich
D7	Ethyl nonanoate	1535.00	A	0.98	Sigma–Aldrich
D8	Ethyl decanoate	1639.00	A	0.99	Sigma–Aldrich
D9	Ethyl 2-hydroxy-4-methylpentanoate	1547.00	B		
D10	Ethyl furoate	1623.00	B		
D11	Ethyl benzoate	1678.00	B		
D12	Ethyl undecanoate	1725.00	B		
D13	Ethyl 9-decenoate	1675.00	B		

D14	Ethyl phenylacetate	1798.00	B		
D15	Ethyl dodecanoate	1848.00	A	0.98	Sigma–Aldrich
D16	Ethyl dihydrocinnamate	1893.00	B		
D17	Ethyl myristate	2038.00	B		
D18	Ethyl palmitate	2263.00	B		
D19	Diethyl succinate	1682.00	A	0.99	Sigma–Aldrich
	Other esters				
E1	Methyl octanoate	1390.00	A	0.99	Sigma–Aldrich
E2	Isoamyl hexanoate	1458.00	A	0.98	Sigma–Aldrich
E3	Isobutyl hexanoate	1350.00	B		
E4	Methyl salicylate	1792.00	A	0.99	Sigma–Aldrich
E5	Propyl octanoate	1513.00	B		
E6	Isoamyl octanoate	1659.00	B		
E7	Isobutyl octanoate	1545.00	B		
E8	Isoamyl lactate	1573.00	B		
E9	Methyl decanoate	1586.00	B		
E10	Methyl laurate	1791.00	B		
E11	Isopentyl decanoate	1840.00	B		
	Fatty acids				
F1	Propanoic acid	1551.00	A	0.99	Sigma–Aldrich
F2	Isobutyric acid	1590.00	A	0.99	Sigma–Aldrich
F3	Isovaleric acid	1683.00	A	0.99	Sigma–Aldrich
F4	Hexanoic acid	1860.00	A	0.99	Sigma–Aldrich
F5	Octanoic acid	2075.00	A	0.99	Sigma–Aldrich
F6	Butanoic acid	1620.00	B		
F7	Decanoic acid	2292.00	A	0.98	Sigma–Aldrich
	Terpenes				
G1	*cis*-Rose oxide	1356.00	A	0.99	Sigma–Aldrich
G2	Linalool	1547.00	A	0.97	Sigma–Aldrich
G3	Citronellyl acetate	1658.00	B		
G4	Citronellol	1770.00	A	0.95	Sigma–Aldrich
G5	Nerol	1805.00	A	0.97	Sigma–Aldrich
G6	α-Terpineol	1703.00	A	0.99	Sigma–Aldrich
G7	Methyl geranate	1686.00	B		
G8	Geranylacetone	1864.00	A	0.95	Sigma–Aldrich
G9	Geraniol	1855.00	A	0.99	Sigma–Aldrich
	Norisoprenoids				
H1	β-Damascenone	1833.00	B		
H2	Riesling acetal	1555.00	B		
H3	Vitispirane A	1530.00	B		
H4	Vitispirane B	1535.00	B		
H5	TDN	1735.00	B		
	Volatile phenols				
I1	Guaiacol	1862.00	A	0.98	Sigma–Aldrich
I2	4-Methylguaiacol	1933.00	B		
I3	*o*-Cresol	2025.00	A	0.99	Sigma–Aldrich

I4	Phenol	2029.00	A	0.99	Sigma–Aldrich
I5	4-Ethylguaiacol	2031.00	A	0.98	Sigma–Aldrich
I6	*p*-Cresol	2053.00	A	0.99	Sigma–Aldrich
I7	*m*-Cresol	2094.00	A	0.99	Sigma–Aldrich
I8	4-Propylguaiacol	2123.00	B		
I9	Eugenol	2168.00	A	0.99	Sigma–Aldrich
I10	4-Ethylphenol	2198.00	A	0.99	Sigma–Aldrich
I11	4-Vinylguaiacol	2180.00	B		
I12	*cis*-Isoeugenol	2269.00	A	0.98	Sigma–Aldrich
I13	*trans*-Isoeugenol	2342.00	A	0.98	Sigma–Aldrich
I14	4-Vinylphenol	2223.00	B		
I15	Syringol	2251.00	B		
	Phenolic aldehydes				
J1	Vanillin	2566.00	A	0.98	Sigma–Aldrich
J2	Acetovanilone	2640.00	B		
J3	Syringaldehyde	2907.00	A	0.99	Sigma–Aldrich
J4	Acetosyringone	2350.00	B		
	Furanic compounds				
K1	Furfural	1452.00	A	0.99	Sigma–Aldrich
K2	5-Methyl furfural	1570.00	A	0.99	Sigma–Aldrich
K3	Acetylfuran	1508.00	A	0.99	Sigma–Aldrich
K4	Maltol	1973.00	B		
K5	Cyclotene	1806.00	B		
	Oak lactones				
L1	*trans*-Whiskey lactone	1679.00	A	0.98	Sigma–Aldrich
L2	*cis*-Whiskey lactone	1957.00	A	0.98	Sigma–Aldrich
	Carbonyl compounds				
M1	Acetoin	1298.00	A	0.96	Sigma–Aldrich
M2	Benzaldehyde	1534.00	A	0.99	Sigma–Aldrich
M3	Benzeneacetaldehyde	1561.00	A	0.99	Sigma–Aldrich
M4	Decanal	1499.00	A	0.98	Sigma–Aldrich
	Benzenes				
N1	Styrene	1263.00	B		
N2	Naphthalene	1756.00	B		
N3	1-Methylnaphthalene	1890.00	B		
	Others				
O1	Methionol	1726.00	B		
O2	3-Isobutyl-2-methoxypyrazine	1525.00	A	0.99	Sigma–Aldrich

[a] Retention indices on HP-Innowax column.

[b] Identification: (A) volatile identified by mass spectrum and retention indices of reference standard; (B) volatile tentatively identified by mass spectrum and retention indices with literatures.
